# Alternative 5’ Untranslated Regions Are Involved in Expression Regulation of Human Heme Oxygenase-1

**DOI:** 10.1371/journal.pone.0077224

**Published:** 2013-10-02

**Authors:** Marcel Kramer, Christoph Sponholz, Monique Slaba, Bianka Wissuwa, Ralf A. Claus, Uwe Menzel, Klaus Huse, Matthias Platzer, Michael Bauer

**Affiliations:** 1 Integrated Research and Treatment Center, Center for Sepsis Control and Care (CSCC), Jena University Hospital, Jena, Germany; 2 Genome Analysis, Leibniz Institute for Age Research - Fritz Lipmann Institute, Jena, Germany; 3 Department of Anaesthesiology and Intensive Care Therapy, Jena University Hospital, Jena, Germany; 4 Hans Knöll Institute for Natural Product Research and Infection Biology, Leibniz Institute, Jena, Germany; University Hospital S. Maria della Misericordia, Udine, Italy

## Abstract

The single nucleotide polymorphism rs2071746 and a (GT)_n_ microsatellite within the human gene encoding heme oxygenase-1 (*HMOX1*) are associated with incidence or outcome in a variety of diseases. Most of these associations involve either release of heme or oxidative stress. Both polymorphisms are localized in the promoter region, but previously reported correlations with heme oxygenase-1 expression remain not coherent. This ambiguity suggests a more complex organization of the 5’ gene region which we sought to investigate more fully.

We evaluated the 5‘ end of *HMOX1* and found a novel first exon 1a placing the two previously reported polymorphisms in intronic or exonic positions within the 5’ untranslated region respectively. Expression of exon 1a can be induced in HepG2 hepatoma cells by hemin and is a repressor of heme oxygenase-1 translation as shown by luciferase reporter assays. Moreover, minigene approaches revealed that the quantitative outcome of alternative splicing within the 5’ untranslated region is affected by the (GT)_n_ microsatellite.

This data supporting an extended *HMOX1* gene model and provide further insights into expression regulation of heme oxygenase-1. Alternative splicing within the *HMOX1* 5' untranslated region contributes to translational regulation and is a mechanistic feature involved in the interplay between genetic variations, heme oxygenase-1 expression and disease outcome.

## Introduction

Heme oxygenases (HO) break down heme, the oxygen-carrying constituent of red blood cells, yielding biliverdin, iron (II) ions, and carbon monoxide. Among the known human/mammalian/vertebrate isoenzymes, only heme oxygenase-1 (HO-1) can be induced by a panoply of stimuli linked by their ability to provoke oxidative stress [1,2]. HO-1 induction protects against cell death in experimental models associated with ischemia/reperfusion or inflammation, making the gene a promising target in diverse clinical phenotypes, such as myocardial infarction, stroke, or sepsis [3]. Moreover, increasing HO-1 activity might increase stress tolerance, most notably in organ transplantation [4,5]. Induction of the gene encoding HO-1 (*HMOX1*) may be beneficial by regulating intracellular levels of toxic heme or by increased anti-inflammatory, anti-apoptotic, and blood flow-maintaining heme degradation products biliverdin and carbon monoxide. Although protective effects of HO-1 up-regulation have been reported for a variety of cells and tissues, experimental evidence suggests that the protective action may be restricted to a narrow threshold of over-expression, supporting the need for a closer examination of gene expression. There exists ample evidence supporting a role for genetic variation of human *HMOX1* to impact outcome. In particular, the single nucleotide polymorphism (SNP) rs2071746 and a microsatellite (GT-dinucleotide repeat, (GT)_n_ ) in the promoter region of the gene are associated with incidence, progression, or outcome of various clinical diagnoses as diabetes, sepsis, ARDS, myocardial infarction, and failure of kidney and liver grafts [6–10]. These polymorphisms might be an intrinsic component of the pathogenesis of some of these cases via *HMOX1* transcription and/or translation regulation and subsequent HO-1 activity [3].

The (GT)_n_ as well as rs2071746 are located in the promoter region of *HMOX1* (Ref_mRNA NM_002133), which is currently annotated as 5-exon gene on human chromosome 22q12 [11,12]. Both polymorphisms are in strong linkage disequilibrium [10,13]. The pattern of the (GT)_n_ distribution has been shown to be trimodal with 23, 30 and 37 repeats (named here as N23, N30, N37) as major alleles within the three length classes [10]. Although there have been correlations between (GT)_n_ length and HO-1 expression reported, these data remain not coherent. In cultured human lymphoblastoid cells, baseline mRNA levels and baseline HO-1 activity were similar in cells homozygous for short or long (GT)_n_ alleles [14]. When these cells are stimulated with hydrogen peroxide, HO-1 mRNA level and activity were significantly higher for short (GT)_n_ . Interestingly, healthy humans homozygous for long (GT)_n_ alleles showed significantly higher mRNA levels after haem arginate infusion compared to those with short ones [15]. However, no differences between the two groups were seen on protein level, indicating an allele-specific post-transcriptional control. The differences in basal and induced mRNA and protein expression suggest the existence of heme-induced allele-specific transcript isoforms exhibiting different translational efficiency. Moreover, *HMOX1* promoter studies show further inconsistencies. Some studies have reported differences in transcriptional activity for rs2071746 or (GT)_n_ alleles [14,16,17], but Tanaka et al. could not detect any significant difference for these polymorphisms using luciferase reporter assays [13]. Since reporter constructs with truncated promoter sequence do not replicate molecular phenotypes of the endogenous gene, the regulation of the *HMOX1* expression seems to be far more complex than previously believed. Additional regulatory sequence elements upstream of the transcription start site (TSS) and/or alternative 5’ untranslated regions (UTRs) might explain the discrepancies among reports on *HMOX1* expression.

A genome-wide screen for alternative splicing and differential transcription initiation has estimated that a significant number of genes in eukaryotes are differentially spliced within UTRs [18]. Alternative splicing of the 5’ UTR is known to impact mRNA stability and translation (e.g. for the human genes *NOD2* and *SFTPA1/2* [19,20]). Moreover, individual expression differences caused by common polymorphisms frequently result from changes in alternative splicing [21].

In this study, we analyzed potential alternative 5’ UTR splicing in *HMOX1* and its downstream effects on HO-1 expression. We provide evidence for a revised gene structure implicating alternative splicing in the 5’ UTR. Correlation of the polymorphisms rs2071746 and (GT)_n_ with alternative splicing was evaluated using minigenes. Implications of the alternative splice variants on translation efficiency were validated with a luciferase reporter assay.

## Material and Methods

### *HMOX1* minigenes

The sequence of *HMOX1* minigenes correspond to the genomic region between the novel exon 1a and the annotated (e.g. in the UCSC Genome Browser, http://genome.ucsc.edu) second exon of the human gene *HMOX1* with partially shortening of the annotated intron 1. DNA fragments were amplified from a human genomic DNA pool (Roche) using Bioline Biomix (Bioline) and the primers 5’-CGGTTTCCCCATCTGTAAAATAG-3’ and 5’-TTCCTCCCTCCAACTACCCT-3’ as well as 5’-CCAAAGCGCTGGGATTACAG-3’ and 5’-ACCTGGCCCTTCTGAAAGTT-3’. PCR fragments were connected with a linker oligonucleotide 5’-AGGGTAGTTGGAGGGAGGAACCAAAGCGCTGGGATTACAG-3’ in a second PCR. For expression in mammalian cells PCR products were cloned into pcDNA3.3-TOPO expression vector (Invitrogen). After sequence verification, minigenes with required haplotypes were selected and applied for transfection in human cell lines.

### Cell culture and transfection

The human hepatoma cell line HepG2 (ATCC) was cultured in DMEM Ham’s F12 medium (Gibco) supplemented with 10% fetal bovine serum (FBS, PAA Laboratories) and 2 mM L-glutamine (Gibco) at 37°C, 5% CO_2_ and 95% humidity. The human embryonic kidney cell line HEK293-EBNA (Invitrogen) was cultured under equal atmospheric conditions using DMEM high glucose medium (Gibco) supplemented with 10% FBS. Transfection of HepG2 cells was carried out with FuGene HD (Roche) according to manufacturer’s instructions. HEK293-EBNA cells were transfected with Lipofectamine 2000 (Invitrogen) as described in the manufacturer’s protocol. Twenty-four hours after transfection, total RNA was isolated with RNeasy Mini Kit (Qiagen, Hilden, Germany). cDNA was reverse transcribed from 5 µg total RNA using Sprint-RT Random Hexamer First-strand cDNA Synthesis Kit (Clontech-Takara). The effect of hemin on minigene splicing was tested using cells transfected with the *HMOX1* minigene containing the haplotype N30/A. Four hours after transfection procedure cells were treated with 10 µM hemin (in 0.1 M NaOH, Sigma-Aldrich) for 20 h.

### Capillary electrophoresis with laser-induced fluorescence detection (CE-LIF)

Detection and quantification of minigene derived transcripts was done as previously described [22]. Briefly, PCR amplification was carried out under standardized conditions using Bioline BioMix white (Bioline), one plasmid specific 5’-6-carboxyfluorescein (FAM)-labeled primer (5’-FAM-CTCCGGACTCTAGAGGATC-3’) and one *HMOX1* specific primer (5’-ACCTGGCCCTTCTGAAAGTT-3’) according to manufacturer’s instructions. PCR fragments were separated on a capillary sequencer and analyzed with the GeneMapper Software (Applied Biosystems). Comparisons of splice-isoform proportions were made by calculation of percent fractions.

### 5’-RACE

Rapid Amplification of cDNA Ends (RACE) was used to analyze the 5’ end of *HMOX1*. Human Marathon-Ready cDNA from liver, kidney and leukocytes (Clontech-Takara) was amplified with Bioline Biomix and the primers AP-1 (supplied with Marathon-Ready cDNA, Clontech-Takara) and 5’-ACCTGGCCCTTCTGAAAGTT-3’. 5’-RACE products were cloned into pCR2.1TOPO vector using the Topo TA Cloning Kit (Invitrogen) and sequence analyzed. In general, sequencing was done with ABIs BigDye V3.1 chemisty (Applied Biosystems). Sequence analysis was performed on an ABI 3730xl capillary sequencer.

### Reverse transcription (RT)-PCR and sequence analysis

Amplification of endogenous *HMOX1* transcripts was done with nested PCR on cDNA of hemin stimulated HepG2-cells. Bioline BioMix (Bioline) and two sets of primers were used under standard PCR conditions: 5’-CTCGGTTTCCCCATCTGTAAAA-3’ and 5’-GCCAGAGCTGGGCAGGTC-3’ as well as 5’-AGAAGCGATCTACCCTCACAG-3’ and 5’-ACCTGGCCCTTCTGAAAGTT-3’ for nested PCR. Amplification and sequence verification of minigene transcripts was carried out under the same PCR conditions used for laser-induced fluorescence analysis, but without FAM-labeled primers. All PCR products were subcloned and sequence verified as previously described.

### Real Time PCR

Real Time PCR was performed on a Rotor-Gene rotary analyzer (Corbett Life Science) using the Brillant II SYBR Green qPCR Master Mix (Stratagene) according to the manufacturer’s instructions. Primers for *HMOX1* were 5’-CAGTGCCACCAAGTTCAAGC-3’ as well as 5’-GTTGAGCAGGAACGCAgtCTT-3’ and for human beta actin (*ACTB*) 5’-GGCATGGGTCAGAAGGATT-3’ and 5’-AGGTGTGGTGCCAGATTTTC-3’. *HMOX1* mRNA expression levels were normalized to *ACTB* and given as values relative to mock controls.

### Luciferase reporter assay

*HMOX1* 5’ UTR fragments were PCR amplified with Pfu/Psp DNA polymerases premix (GeneOn GmbH) from the respective minigenes. The forward primer 5’-CGGTTTCCCCATCTGTAAAATAG-3’ was combined with the reverse primer 5’-GGACGCTCCATGGGGCCGGTGCTGG-3’ or 5’-CCTGGGCCATGGTGTGAGGGTAGATC-3’ (both providing NcoI restriction site) depending on the transcript of interest. The 5’ ends of the PCR products containing exon 1a sequence were elongated with the synthesized DNA fragment 5’-CTTAATCAAGCTTCGGGGACTTAGTCTCCTCCCTGGGTTTGGACACTGGCATCCTGCTTTATGTGTGACACCACTGCACCCCTCTGAGCCTCGGTTTCCCCATCTGTAAAATAG-3’ (Metabion) performing an overlap extension PCR. The primer 5’-CTTAATCAAGCTTCGGGGACT-3’ was either combined with 5’-GGACGCTCCATGGGGCCGGTGCTGG-3’ for exon 1 or with 5’-CCTGGGCCATGGTGTGAGGGTAGATC-3’ for exon 2 (both containing a HindII restriction site). For the construct containing only exon 1 the primers 5’-CGCCAAGCTTCTCTCGAGCGTCCTC-3’ and 5’-GGACGCTCCATGGGGCCGGTGCTGG-3’ were used without overlap extension. Final PCR products and the luciferase reporter vector pGL3-Promoter (Promega) were digested with HindIII and NcoI (New England Biolabs) according to the supplier’s recommendations. These PCR products were ligated into pGL3-Promoter vector via HindIII and NcoI restriction sites using T4 DNA Ligase (New England Biolabs). Sequences upstream of the ATG start codon of the luciferase gene (NcoI restriction site) were set back to endogenous sequence with the Quik Change XL site-directed mutagenesis kit (Stratagene) and the primers 5'-CCAGCACCGGCCGGATGGAAGACGCCAAAAAC-3' and 5'-GTTTTTGGCGTCTTCCATCCGGCCGGTGCTGG-3' for exon 1 as well as 5'-CTGTAAAATAGAAGCGATCTACCCTCACAGCATGGAAGACGCC-3' and 5'-GGCGTCTTCCATGCTGTGAGGGTAGATCGCTTCTATTTTACAG-3' for exon 2. All plasmid constructs were verified by sequencing.

Twenty-four hours before transfection HepG2 and HEK293-EBNA were seeded in 96-well culture plates with 2.0 x 10^4^ or 2.5 x 10^4^ cells respectively. HepG2 cells were co-transfected with Fugene HD Transfection Reagent (Roche) according to manufacturer’s instructions using 0.12 µg experimental DNA derived from the pGL3 promoter vector and 0,0025 µg pRL-SV40 vector (Promega, ratio 1:48). Co-transfection of HEK293-EBNA cells was performed with 0.15 µg experimental DNA derived from the pGL3 promoter vector and 0,015 µg pRL-SV40 vector (Promega, ratio 1:10) and Lipofectamine (Invitrogen) following the manufacturer’s protocol. After 24 h of incubation luminescence measurements were done with the Dual-Glo Luciferase Assay System (Promega) and the *Mithras* LB 940 plate reader (Berthold Technologies GmbH & Co KG). Renilla expressed by the pRL-SV40 vector (Promega) served as internal standard for firefly activity of the 5’ UTR reporter plasmid (derived from pGL3 promoter vector). Transfections were performed in three independent experiments with three replicates each. Firefly/renilla ratios were background subtracted and given as relative luciferase units (RLU) relative to unmodified pGL3 promoter vector.

### Immunoblotting

HepG2 cells were lysed in STEN lysis buffer (50 mM Tris pH 7.6, 150 mM NaCl, 2 mM EDTA, 1% NP40 and protease inhibitor) for 30 min on ice. After centrifugation the protein concentration in the supernatant was measured with the Pierce BCA Protein Assay Kit (Thermo, Fisher Scientific) according to manufacturer’s instructions. 25 µg protein were heat denatured in SDS loading buffer (65 °C, 10 min). Polyacrylamide gel electrophoresis (PAGE) in NuPAGE Novex 10% Bis-Tris gels (Invitrogen) and Western Blotting on PVDF membrane was carried out with XCell SureLock Mini-Cell and XCell II Blot Module from Invitrogen. For immunoprecipitation, we used anti-heme oxygenase 1 mouse monoclonal (ab13248) and anti-beta actin rabbit polyclonal (ab8227) antibodies from Abcam (Abcam). HRP conjugated anti-mouse IgG (W4021) and anti-rabbit IgG (W4011) antibodies were supplied by Promega.

### Hemin treatment

HepG2 cells were treated with 10 µM hemin (in 0.1 M NaOH, Sigma-Aldrich) for 6 h or 24 h. Mock controls were treated with the equal amount of 0.1 NaOH. Subsequently, cells were lysed either to obtain total RNA or protein fractions as previously described.

### Data management and statistical analysis

If not indicated otherwise, all data were obtained from at least three independent experiments and given as mean ± standard deviation. Statistical calculations were done with Sigma Plot 11 (Systat Software) and the R statistical language suite (www.r-project.org).

## Results

### *HMOX1* gene structure revised: additional upstream exon and 5’ UTR alternative splicing

*In silico* analyses using expressed sequence tags (EST, BE407102) indicate a novel first exon 1a (GRCh37/hg19, chr22:35,776,354-35,776,473) in the 5’ UTR of *HMOX1*. Identification of this exon places the polymorphisms rs2071746 and (GT)_n_ in intronic positions. Exon 1a originates from a mammalian-wide interspersed repeat (MIRb, chr22:35776335-35776503). In addition, EST DA903962 supports an extended 5' UTR, resulting from an alternative splicing event retaining the (GT)_n_ in the first internal exon. Such an intronic or exonic location would have consequences for the functional estimations of both polymorphisms which hitherto have solely been considered as promoter polymorphisms.

**Figure 1:**
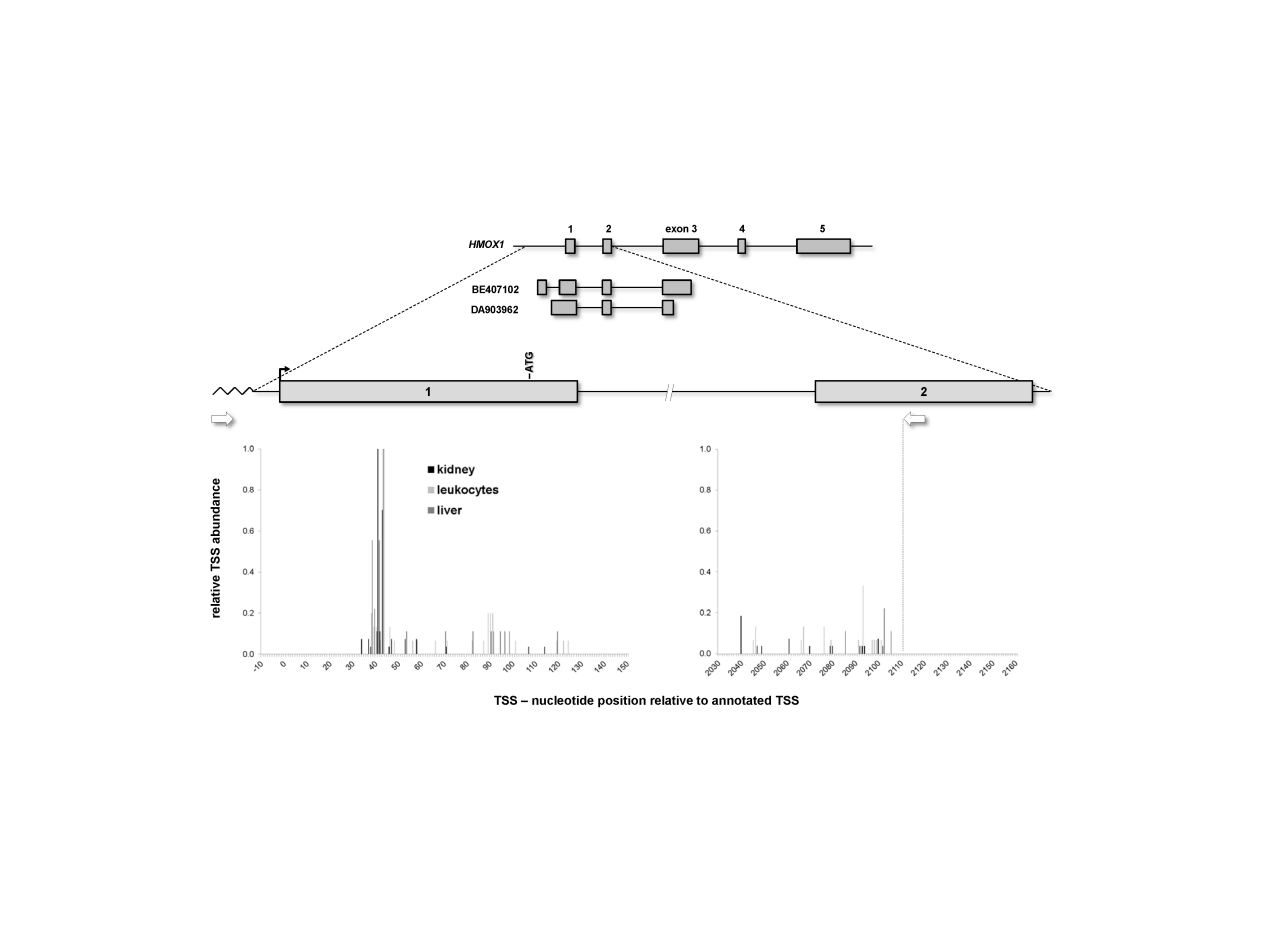
*HMOX1* transcription start site usage in human tissues. Graphical representation of the gene *HMOX1* on human chromosome 22q12 and the two expressed sequence tags BE407102 and DA903962. Boxes represent exonic regions and constant **lines** intronic sequence. 5’ RACE was performed on human cDNA tissue panels. RACE primers are indicated by white arrows. Transcription start sites (TSSs) represent the first nucleotide of the transcripts. TSS positions are given as nucleotides relative to the annotated TSS (GRCh37/hg19, chr22:35,777,060) at zero (black arrow). The canonical translation start codon in exon 1 is indicated by ATG.

**Figure 2:**
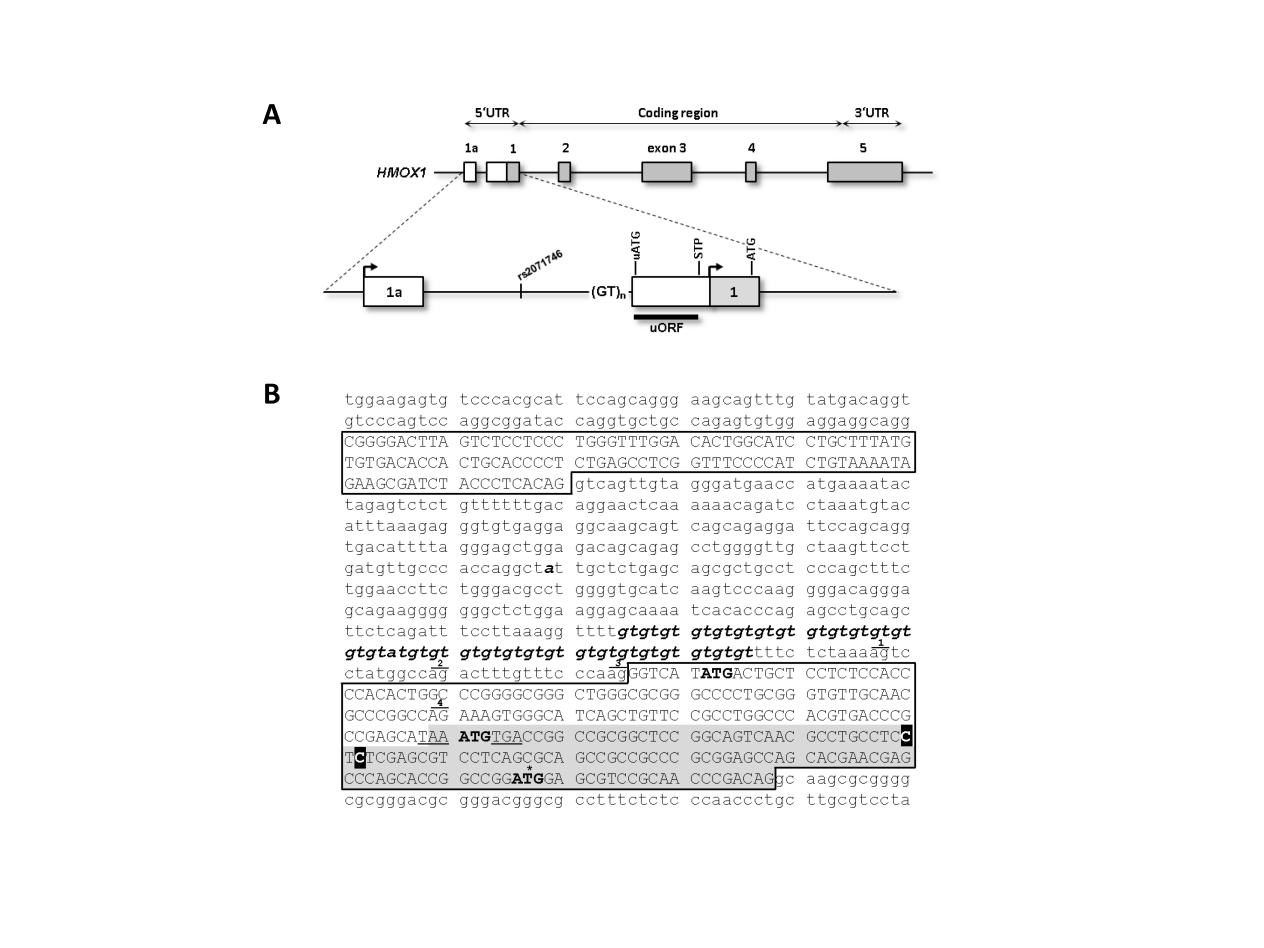
Re-evaluated 5’ region of the human gene *HMOX1*. (**A**) Exon-intron structure of *HMOX1*. Exons are represented by boxes, whereas white boxes represent novel exonic region. The two independent transcription initiation sites are indicated by black arrows. ATG is the translation start codon of the HO-1 coding region. uATG and downstream STP (stop codon) define the upstream open reading frame (uORF). (**B**) Sequence of the *HMOX1* 5’ region. Exons are represented by capitalized letters and the annotated first exon is highlighted by a grey box. Bolt italic letters correspond to the SNP rs2071745 and the (GT)_n_ microsatellite. The first nucleotide of the common transcription start sites are boxed in black. Alternative splice acceptor sites (AG1-AG4) at exon 1 are numbered above. Translation start codons are in bold letters and stop codons are underlined. The canonical ATG is marked by an asterisk.

**Figure 3:**
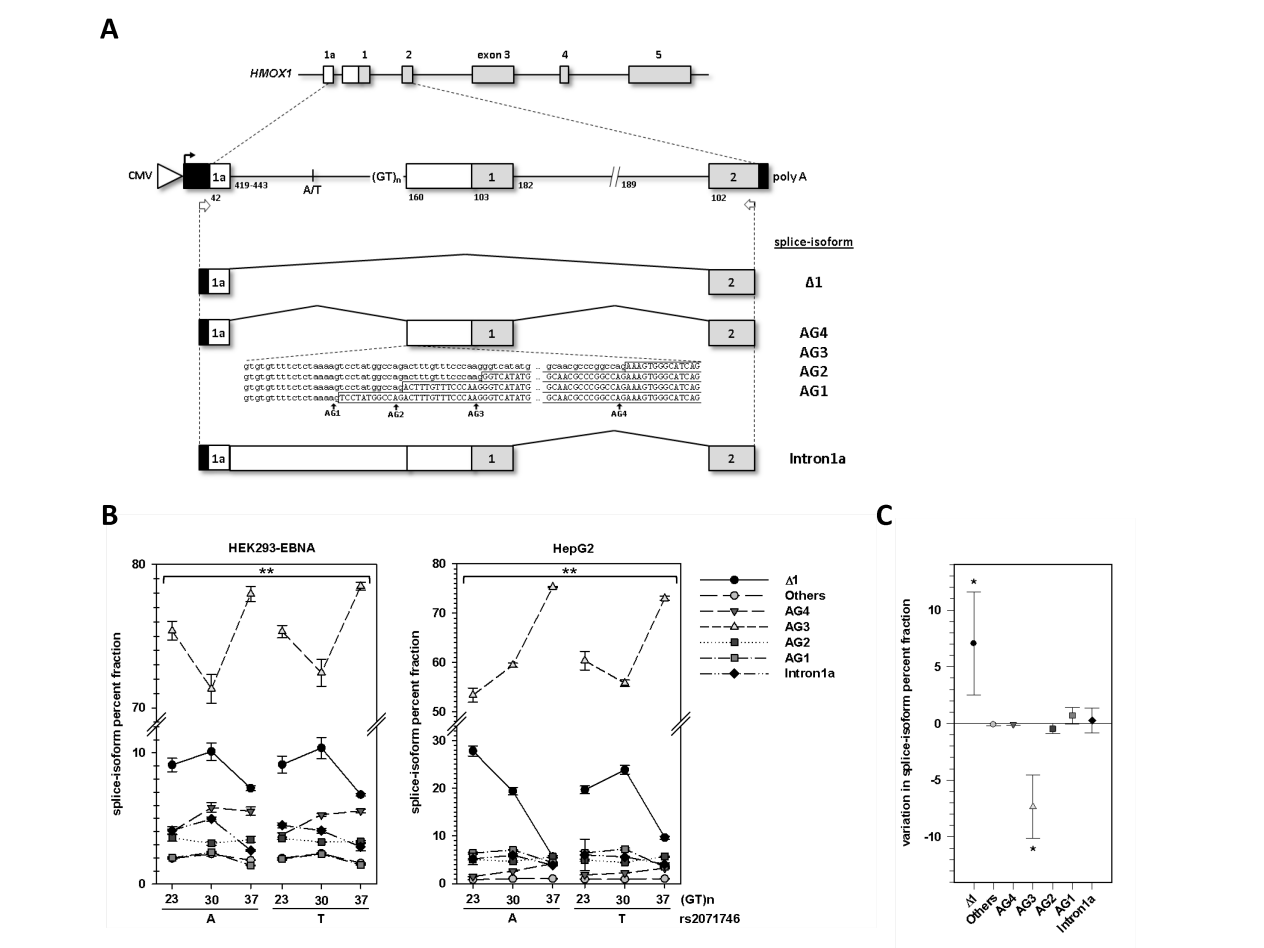
Polymorphism-dependent alternative splicing at the *HMOX1* 5’ region. (**A**) Schematic representation of the *HMOX1* minigene and expressed splice-isoforms. Boxes represent exonic regions and constant **lines** intronic sequence. Black boxes are exonic sequences provided by the pcDNA3.3 plasmid. The CMV promoter is represented by a triangle. Given numbers correspond to nucleotide length. Primers for RT-PCR are indicated by white arrows. (**B**) The amount of *HMOX1* minigene splice-isoforms found in HEK293-EBNA and HepG2 cells are given as percent fractions (means of n=3 ± standard deviation). The significance level for the differences in the splice-isoform profiles between the six haplotypes was calculated with the G-test (**p<0.001). (**C**) Changes in *HMOX1* minigene (N30/A) percent splice-isoform fractions in HepG2 cells treated with 10µM hemin for 20 hours. Data are plotted as means (n=3) ± standard deviation. Statistical significance was calculated with respect to the control using t-test (*p<0.05).

To experimentally characterize the *HMOX1* gene structure upstream of the annotated exon **1** (chr22:35,777,060-35,777,189) in more detail we performed 5’ RACEs on human tissue cDNA panels from leukocytes, kidney and liver. Although we were able to find a multitude of TSSs in exon 1 and exon 2 (Figure 1), all were located downstream of the annotated TSS. Interestingly, TSSs clustered in all three tissues at two spots (+41 and +43 nucleotides). Although the respective transcript contain the canonical translation initiation codon, our findings put the current annotation of the 5’ end of exon 1 into question, but 5’ RACE products failed to extend into exon 1a. Since *HMOX1* has a very low basal expression in the tested cDNA panels, we performed nested RT-PCR in liver-derived HepG2 cells stimulated with 10 µM hemin for 24 h, which massively induced HO-1 mRNA and protein expression (Figure S1). Placing primers on exon 1a from BE407102 and on the canonical exon 2 revealed a number of novel splice variants (Figure S2) including several 5’ elongations of exon 1 (alternative splice acceptors AG1-AG4, Figures 2B and 3A), exon 1 skipping (Δ1) or intron 1a retention (Intron1a). All alternative exon 1 splice acceptors were found downstream of the (GT)_n_ microsatellite. The alternative transcript utilizing the splice acceptors AG1-AG3 (Figure 3A) contain an upstream open reading frame (uORF, Figure 2). The stop codon of the uORF is followed by an additional start stop codon sequence. Splicing of exon 1a to exon 2 (Δ1) results in an in-frame downstream translation initiation site in exon 2. This would truncate the protein at the N-terminus.

### Alternative 5’ UTR splicing in *HMOX1* minigenes

Cis-acting polymorphisms affecting alternative splicing are known phenomena, but are only partially understood. Both rs2071746 and (GT)_n_ variants influence *HMOX1* mRNA and protein expression and can be located in a primary transcript as alternative intronic or exonic elements. We therefore analyzed the splice-regulatory potential of these variants by establishing minigenes containing the genomic sequence of human *HMOX1* ranging from exon 1a to exon 2. Intron 1 (1,9 kb in size) was shortened for practicality reasons, retaining 182 and 189 nucleotides, respectively, of the intron flanks (Figure 3A). Six minigenes were constructed representing the three major alleles of (GT)_n_ length groups (N23; N30; N37) each with either the T or A allele of rs2071746. All minigene transcripts were sequenced and the splice-isoforms were quantified by fluorescence RT-PCR and capillary electrophoresis with laser-induced fluorescence detection (CE-LIF). All minigenes (haplotypes) tested, showed the same splice-isoforms even in the two different cell lines (HepG2, HEK293-EBNA) transfected. The minigene splice-isoforms (Figure 3A) detected also corresponded to those found endogenously in HepG2 cells (Figure S2). Only the AG2 splice-isoform, which is of minor frequency in minigenes, had not been previously identified endogenously within a set of 131 sequenced HepG2 cDNA clones. The most frequent isoforms were AG3 and Δ1 (Figure 3B). AG3 isoform frequencies among all haplotypes varied between 71.3 (±1.0)% and 78.5 (±0.3)% in HEK293-EBNA cells and between 53.4 (±1.0)% to 75.3 (±1.6)% in HepG2 cells. The second most common isoform was Δ1 with a range of 5.7 (±0.2)% and 27.8 (±1.3)% in HepG2 cells and between 6.8 (±0.1)% and 10.4 (±0.8)% in HEK293-EBNA cells. The most conspicuous effects on splice-isoform frequencies were related to the (GT)_n_ length. In both cell lines, N37 minigenes produced the highest levels of the AG3 isoform and the lowest levels of Δ1, independent of the rs2071746 allele and the reverse found for N30. N23 showed intermediate splice-isoform frequencies, with the minigene representing the haplotype N23/A produced in HepG2 as the sole exception, showing the lowest amount of AG3 isoform and the highest levels of Δ1 among all minigenes tested. Nevertheless, likelihood ratio test confirmed that the splice-isoform distributions of the minigenes were significantly different between all six haplotypes on a 95% confidence level (p<0.001, G-test).

Hemin induces HO-1 mRNA and protein expression in a dose-dependent manner, as it has been shown in rat skeletal muscle cells [23]. We tested, whether hemin also influences splicing by transfecting HepG2 with the *HMOX1* minigene N30/A (Figure 3C). Hemin treatment significantly decreased the fraction of AG3 (-7.3%; p=0.003, t-test), but increased splicing of Δ1 (7.0%, p=0.019, t-test). All other changes were lower than 1%.

### Luciferase reporter assay on *HMOX1* 5’ UTRs

**Figure 4:**
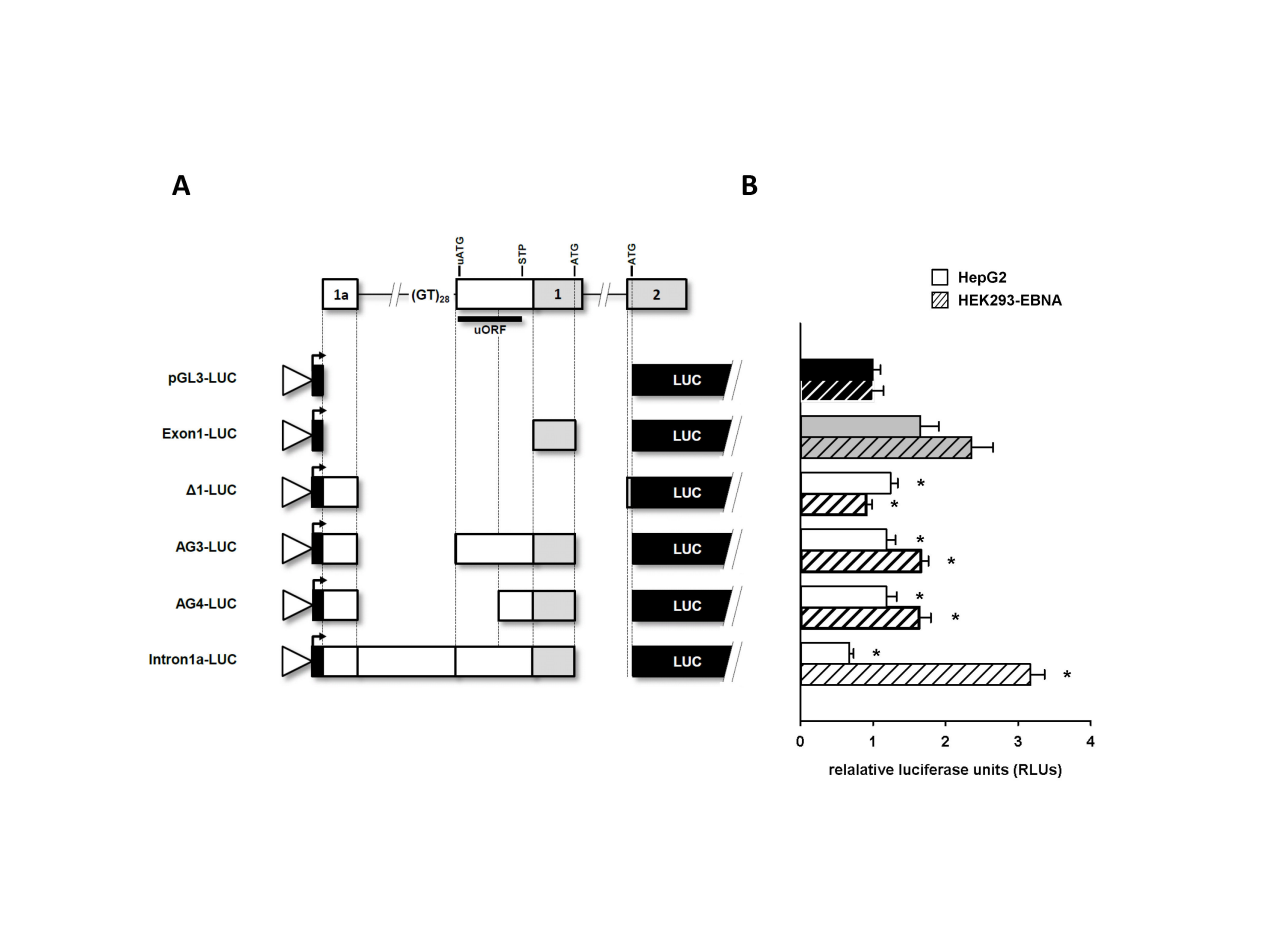
Alternative 5’ UTR splicing effects HO-1 translation efficiency. (**A**) Schematic representation of the *HMOX1* 5’ UTR luciferase gene reporter constructs. Boxes represent transcribed regions included in reporter constructs. pGL3 promoter vector contains SV40 promoter (white triangle), transcription start site (black arrow) and firefly luciferase gene (black box). *HMOX1* sequence context includes translation initiation codon (ATG) in exon 1 (canonical ATG) and in exon 2 as well as upstream open reading frame features uATG and STP (stop codon). (**B**) Relative luciferase units (RLUs) are given either for HepG2 or HEK293-EBNA cells transfected with the luciferase gene reporter constructs. Values are background subtracted and normalized to non-modified pGL3 promoter vector (pGL3-LUC). Data are plotted as means (n=9) ± standard deviation. Significance levels were calculated versus Exon1-LUC construct for each cell line independently using the Dunnett’s test (* p<0.05).

Using a luciferase reporter assay, translational efficiency of alternative *HMOX1* 5’ UTRs containing exon 1a (AG3, AG4, Δ1, Intron1a) was compared to the 5’ UTR lacking exon 1a and containing the canonical first exon (TSS +41, Figure 4A). Among the five luciferase constructs Exon1-LUC showed the highest relative luciferase units (RLUs, relative to unmodified pGL3 plasmid) with 1.65 (±0.26) in HepG2 cells (Figure 4B). RLUs for AG3-LUC (1.18 ±0.13), AG4-LUC (1.18 ±0.14) and Δ1-LUC (1.24 ±0.10) were lower compared to Exon1-LUC, but all at the similar intensities. Intron1a-LUC showed the lowest translational efficiency in HepG2 cells with 0.67 (±0.06) RLUs and did not exceed the values of the pGL3 plasmid.

Interestingly, in HEK293-EBNA cells, Intron1a-LUC produced the highest luciferase activity 3.17 (±0.20) RLUs. We also measured 2.36 (±0.29) RLUs in these cells for Exon1-LUC and comparable levels of 1.66 (±0.10) RLUs for AG3-LUC and 1.64 (±0.16) RLUs for AG4-LUC, consistent with the results observed in HepG2 cells. RLUs of Δ1-LUC were 0.91 (±0.08), lower than in the HepG2 cells. Alternative 5’ UTRs had significantly lower translation efficiency compared to the 5’ UTR of the canonical first exon in both in HepG2 cells (p<0.05, Dunnett’s test) as well as in HEK293-EBNA cells (p<0.05, Dunnett’s test) with the exception of Intron1a-LUC in HEK293-EBNA cells. Intron1a-LUC had significantly (p<0.05, Dunnett’s test) higher luciferase activity compared to Exon1-LUC. Transcript analysis revealed alternative splicing of Intron1a-LUC 5’ UTR in HepG2 but not in HEK293-EBNA cells (data not shown). Nevertheless, most abundant 5’ UTR splice variants containing the novel first exon 1a (AG3, Δ1) do have functional translation initiation sites, but the translation rates are significantly lower compared to transcripts initiated at the canonical TSS of exon 1.

## Discussion

Genes in the human genome have been comprehensively annotated both during and following the human genome project as detailed knowledge of gene structure is critical for the analysis and interpretation of gene regulation. Nevertheless, annotations of many genes often rely only on predictions or are supported by a low amount of transcript sequence data and must therefore be considered preliminary. Missing data concerning alternative 5’ ends of genes is particularly noteworthy, as transcripts expressed at low levels, in narrow developmental windows and/or under specific physiological conditions are especially difficult to accurately annotate. This difficulty is particularly pronounced when considering that transcript variability at 5’ ends is high due to alternative TSSs (alternative promoters as well as by dynamic usage of TSSs under the control of the same promoter) and alternative routes of transcript maturation (splicing).

This study sought to increase the body of knowledge regarding the 5’ end of *HMOX1*, as knowledge of its complete gene structure and alternative transcripts is crucial to understand HO-1 regulation. 5’ RACE analyses revealed multiple TSSs with clusters located downstream of the annotated TSS, but upstream of the canonical translation initiation codon (Figure 1). No evidence was found by 5’ RACE for a TSS upstream of the first exon which would give rise to an additional first exon (exon 1a). Exon 1a is expected to be located 627 bp (distance between splice donor exon 1a and novel TSS at exon 1) upstream of the canonical first exon (Figure 2), an expectation supported by EST sequences. Because the 5’ RACE was performed in commercially available cDNA tissue panels, only information on the basal expression of the inducible *HMOX1* could reasonably be obtained. To address this limitation, we extended the analysis using an RT-PCR approach to HepG2 cell culture system, as ENCODE RNA-seq tracks in the UCSC genome browser suggested expression of exon 1a transcripts in these cells [24]. *HMOX1* transcript isoforms encoding exon 1a could be detected in HepG2 cells, but only if cells had been stimulated with hemin (Figure S2). Unfortunately, 5’ RACE on transcripts derived from cells stimulated with hemin failed as 5’ RACE requires homopolymeric tailing of the cDNA. Since gene-specific cDNA synthesis was successful in our RT-PCR approach, tailing and/or PCR problems might be generated by the sequence context of exon 1a which is a repetitive sequence element widely distributed throughout the genome. Sequence features allocated by MIRb elements can act as upstream transcriptional promoters and often provide functional splice sites [25,26]. We have previously identified extra evidence for MIRb exonization in the human genome with the functional characterization of a novel *NOD2* splice-isoform [22].

HO-1 induction by hemin requires inactivation of the transcriptional repressor BACH1 by displacement with NFR2 [27]. Respective ChiP-seq enrolled two BACH1/NRF2 enhancer elements around 4 and 9 kb upstream of the canonical first exon. Within 1 kb upstream of the alternative exon 1a transcription factor ChIP-seq data from ENCODE [28] indicate two clusters of transcription factors binding sites. These clusters may indicate the existence of a second promoter, which could be controlled by BACH1/NRF2 as well.

Gene expression is a highly complex series of processes including gene transcription, maturation of primary transcripts, translation and posttranslational modifications. On the level of transcription initiation, sequence variations and functional sequence motifs involved in regulatory processes are likely to be located within regions flanking the TSSs. In the case of *HMOX1*, both polymorphisms investigated in our study are located in a promoter region upstream of major TSSs mapped in this study. These differences in *HMOX1* promoter transcriptional activity could be due to different (GT)_n_ lengths, because the alternating purine-pyrimidine sequence can form thermodynamically unfavorable Z-DNA conformations [29]. Unfortunately, inconsistent classifications of (GT)_n_ alleles and conflicting results concerning the correlations of (GT)_n_ and rs2071746 with HO-1 mRNA and protein levels make an interpretation of published data difficult. Moreover, some *HMOX1* promoter studies have reported variations in transcriptional activity for rs2071746 or (GT)_n_ alleles, whereas others could not detect any significant effect for any of these polymorphism in luciferase reporter assays [13,14,16,17]. This discrepancy might be explained by tissue-specific expression or limitations of the assays used to reproduce a complex endogenous situation. Our extended *HMOX1* gene model provide further insights into the regulation of HO-1 expression, because alternative splicing of 5' UTRs of mammalian genes are likely to contribute to the translational control [30] via modulation of mRNA stability and/or translational efficiency [31]. Primary transcripts initiated by the promoter of exon 1a contain rs2071746 and the microsatellite (GT)_n_ , which in turn affect quantitative outcome of 5’ UTR alternative splicing. Treatment of hepatoma cells (HepG2) with hemin stimulates HO-1 mRNA and protein expression (Figure S1) and affects alternative splicing within the 5’ UTR of *HMOX1* as well (Figure 3C). This observation implies a biological relevance for this process, as *HMOX1* expression requires a stimulus. Moreover, reporter assays showed that 5’ UTRs containing exon 1a or exon 1 differ in their translational efficiency (Figure 4). Surprisingly, plasmids containing intron 1a showed higher translational capacity compared to the 5’ UTR of exon 1 in HEK293-EBNA cells. Although we detected an alternatively spliced reporter transcript, we cannot exclude the possibility of additional promoter activity from the intron 1a sequence increasing transcriptional efficiency of the canonical first exon. Importantly, our findings are able to explain at least some of the conundrums surrounding HO-1 expression, because hemin stimulates expression from the major TSSs and induces expression of 5’ elongated primary transcripts which in turn decrease mature mRNA isoforms translation. For this reason, human HO-1 mRNA and protein levels may fail to correlate as previously reported [15]. Finally, protective effects of HO-1 up-regulation are restricted to a narrow window of over-expression, because metabolites are potentially toxic [3]. For this reason, there must exist a mechanism able to fine-tune HO-1 expression and limit the amount of active enzyme. The data presented here support a model of an intrinsic mechanism able to reduce protein expression based on transcriptional control of an alternative exon and subsequent differential RNA processing.

Elaborating the regulation of HO-1 expression by alternative transcripts is necessary in order to fully understand the endogenous function of this protein. Unfortunately this endeavor is challenged in practical terms by the repetitive origin of exon 1a. Our data clearly demonstrate an additional, heretofore unknown, level of complexity in *HMOX1* expression regulation which should be further investigated given the role of HO-1 in medical conditions associated with heme release or oxidative stress.

## Supporting Information

Figure S1 **HO-1 mRNA and protein levels in HepG2 cell treated with hemin.** HepG2 cells were treated with 10 µM hemin for 6 h and 24 h. (**A**) Representative Western-Blot of HO-1 and corresponding β-actin as load control. (**B**) Relative mRNA levels were obtained by real time PCR and calculated for 6 h and 24 h separately. Data represent the mean (n=3) ± standard deviation.
(TIFF)

Figure S2 **Sequence alignment of *HMOX1* transcripts derived from HepG2 cells stimulated with hemin.** HepG2 cells were treated with 10 µM hemin for 24 h. Transcripts were amplified with a nested PCR approach and subsequently cloned and sequenced. Exons are represented by boxes, whereas grey boxes correspond to the annotated transcription start. Translation start codons are in bold letters and stop codons are underlined. The productive ATG is marked by an asterisk.
(TIFF)
