# Supplementary figures and images for: Alternative 5’ Untranslated Regions Are Involved in Expression Regulation of Human Heme Oxygenase-1
Source: PLoS One. 2013 Oct 2;8(10):e77224. doi: 10.1371/journal.pone.0077224 (PMC3788786; doi:10.1371/journal.pone.0077224)

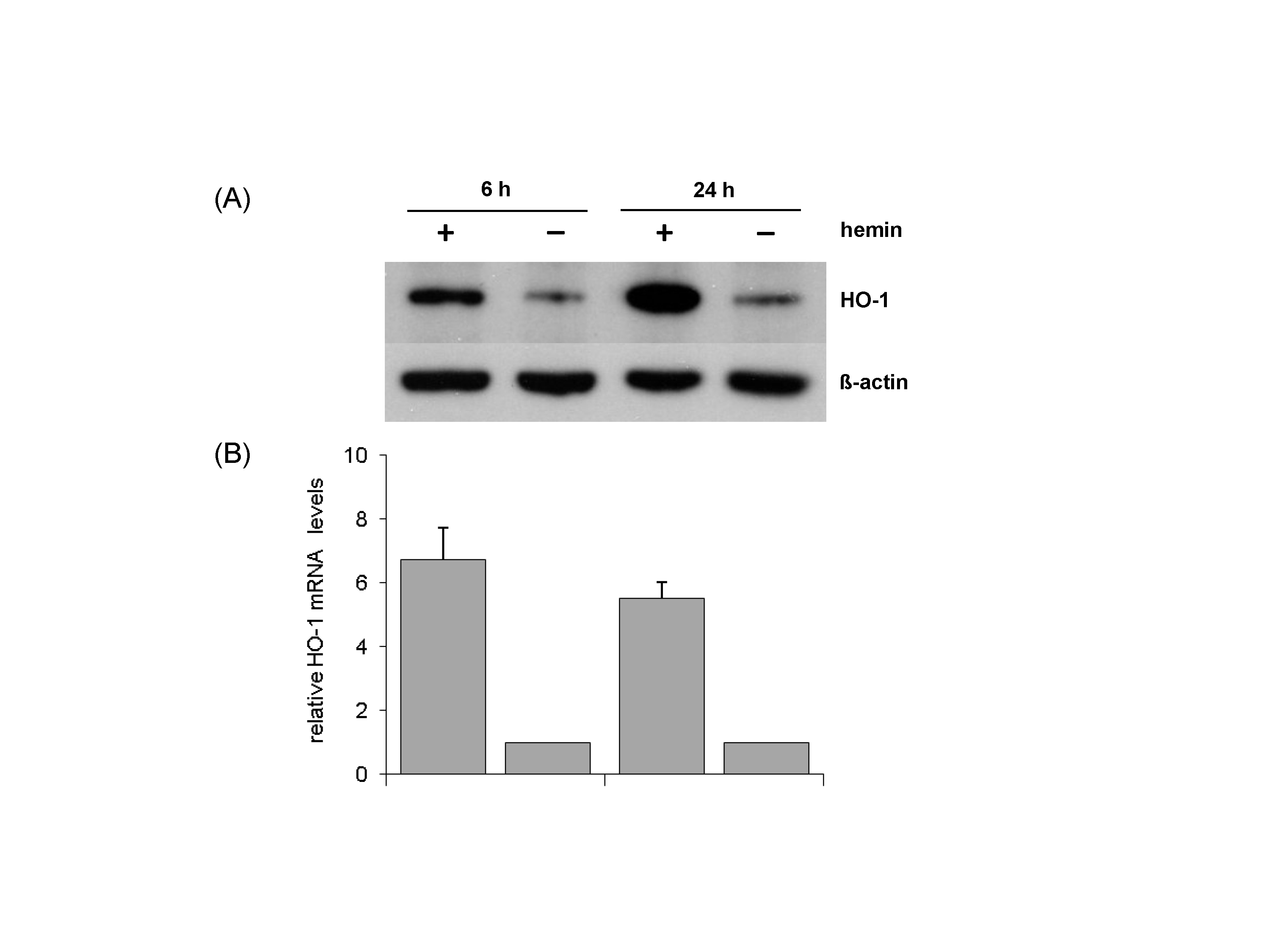

Supplement: Figure S1 — HO-1 mRNA and protein levels in HepG2 cell treated with hemin. HepG2 cells were treated with 10 µM hemin for 6 h and 24 h. (A) Representative Western-Blot of HO-1 and corresponding β-actin as load control. (B) Relative mRNA levels were obtained by real time PCR and calculated for 6 h and 24 h separately. Data represent the mean (n=3) ± standard deviation. (TIFF) [file pone.0077224.s001.tiff]

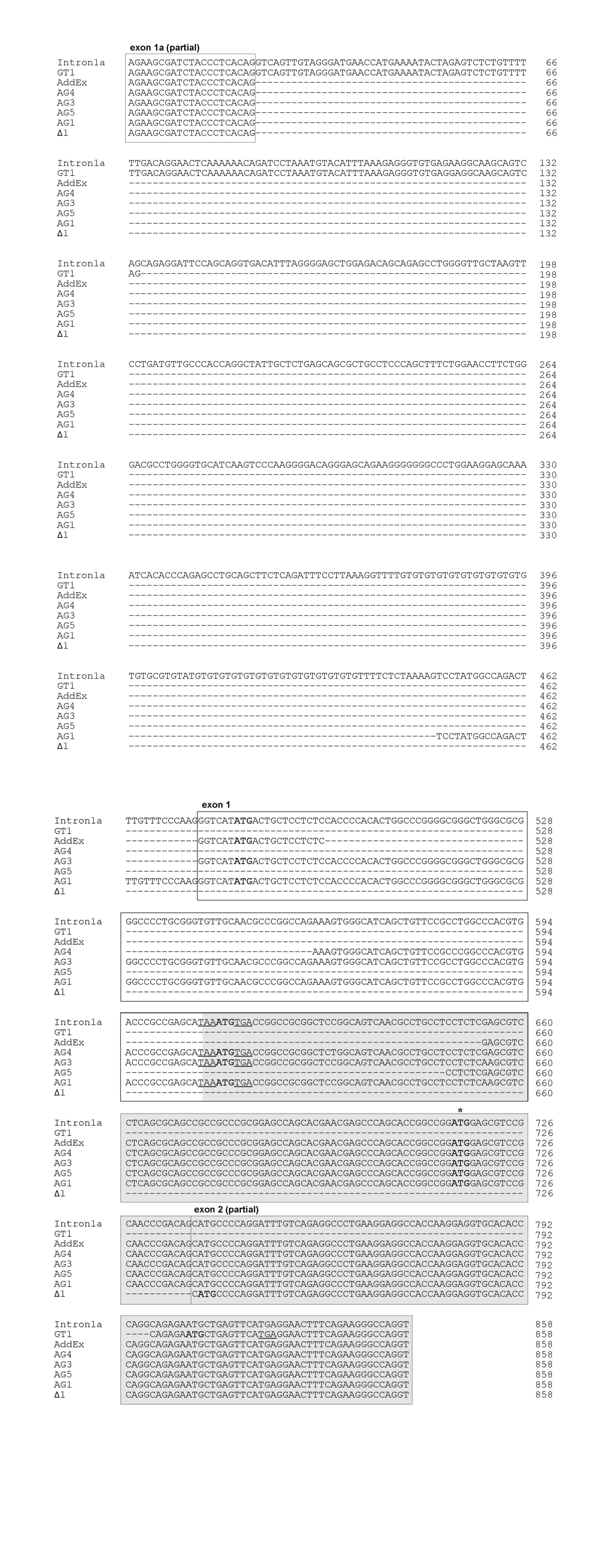

Supplement: Figure S2 — Sequence alignment of HMOX1 transcripts derived from HepG2 cells stimulated with hemin. HepG2 cells were treated with 10 µM hemin for 24 h. Transcripts were amplified with a nested PCR approach and subsequently cloned and sequenced. Exons are represented by boxes, whereas grey boxes correspond to the annotated transcription start. Translation start codons are in bold letters and stop codons are underlined. The productive ATG is marked by an asterisk. (TIFF) [file pone.0077224.s002.tiff]
